# The Granule Size Mediates the In Vivo Foreign Body Response and the Integration Behavior of Bone Substitutes

**DOI:** 10.3390/ma14237372

**Published:** 2021-12-01

**Authors:** Manuel Abels, Said Alkildani, Annica Pröhl, Xin Xiong, Rumen Krastev, Tadas Korzinskas, Sanja Stojanovic, Ole Jung, Stevo Najman, Mike Barbeck

**Affiliations:** 1 BerlinAnalytix GmbH, 12109 Berlin, Germany; manuel.abels@berlinanalytix.com (M.A.); said.alkildani@berlinanalytix.com (S.A.); annica.proehl@berlinanalytix.com (A.P.); 2 Clinic and Policlinic for Dermatology and Venereology, University Medical Center Rostock, 18057 Rostock, Germany; ole.tiberius.jung@gmail.com; 3 NMI Natural and Medical Sciences Institute, University of Tübingen, 72770 Reutlingen, Germany; xin.xiong@nmi.de; 4 Faculty of Applied Chemistry, Reutlingen University, 72762 Reutlingen, Germany; rumen.krastev@reutlingen-university.de; 5 Bokštų Odontologijos Klinika, 92125 Klaipėda, Lithuania; tadaskorzinskas@yahoo.de; 6 Department for Cell and Tissue Engineering, Faculty of Medicine, University of Niš, 18000 Niš, Serbia; s.sanja88@gmail.com (S.S.); stevo.najman@gmail.com (S.N.); 7 Department of Biology and Human Genetics, Faculty of Medicine, University of Niš, 18000 Niš, Serbia; 8 Department of Ceramic Materials, Chair of Advanced Ceramic Materials, Institute for Materials Science and Technologies, Technical University of Berlin, 10623 Berlin, Germany

**Keywords:** bone substitute, bone regeneration, granule size, macrophages, multinucleated giant cells (MNGCs), inflammation, histomorphometry, in vivo

## Abstract

The physicochemical properties of synthetically produced bone substitute materials (BSM) have a major impact on biocompatibility. This affects bony tissue integration, osteoconduction, as well as the degradation pattern and the correlated inflammatory tissue responses including macrophages and multinucleated giant cells (MNGCs). Thus, influencing factors such as size, special surface morphologies, porosity, and interconnectivity have been the subject of extensive research. In the present publication, the influence of the granule size of three identically manufactured bone substitute granules based on the technology of hydroxyapatite (HA)-forming calcium phosphate cements were investigated, which includes the inflammatory response in the surrounding tissue and especially the induction of MNGCs (as a parameter of the material degradation). For the in vivo study, granules of three different size ranges (small = 0.355–0.5 mm; medium = 0.5–1 mm; big = 1–2 mm) were implanted in the subcutaneous connective tissue of 45 male BALB/c mice. At 10, 30, and 60 days *post implantationem*, the materials were explanted and histologically processed. The defect areas were initially examined histopathologically. Furthermore, pro- and anti-inflammatory macrophages were quantified histomorphometrically after their immunohistochemical detection. The number of MNGCs was quantified as well using a histomorphometrical approach. The results showed a granule size-dependent integration behavior. The surrounding granulation tissue has passivated in the groups of the two bigger granules at 60 days *post implantationem* including a fibrotic encapsulation, while a granulation tissue was still present in the group of the small granules indicating an ongoing cell-based degradation process. The histomorphometrical analysis showed that the number of proinflammatory macrophages was significantly increased in the small granules at 60 days *post implantationem*. Similarly, a significant increase of MNGCs was detected in this group at 30 and 60 days *post implantationem.* Based on these data, it can be concluded that the integration and/or degradation behavior of synthetic bone substitutes can be influenced by granule size.

## 1. Introduction

In maxillofacial surgery and dentistry, but also in medical fields such as orthopedics or traumatology, so-called bone substitute materials (BSM) are applied to allow for successful bone tissue regeneration. Thereby, BSM have gained special importance within the last decades as they allow the reconstruction of bone without co-morbidities which are common with autologous grafting [1,2,3]. The heterogeneous group of BSM can initially be divided into natural and synthetic materials. Synthetic materials are becoming a more reliable alternative to natural BSM within the last years [4,5] due to their high availability and tailorability. Although a lot of basic knowledge about the molecular healing pathways mediated by synthetic BSM has been gathered in the last decades, the effects of different material characteristics on (the underlying processes of) bone tissue regeneration are still unclear [1].

For example, the effect of the granule size or diameter onto the molecular basis of tissue repair is only poorly understood. It has already been revealed that small BSM granules seem to induce a higher inflammatory tissue response associated with an increased implantation bed vascularization, which is an important factor of bone tissue repair [6]. However, it was shown that they are much faster resorbed—also in case of xenogeneic BSM that has been shown to be poorly resorbable [7]. In this context, Leiblein et al. analyzed the effects of granule size onto bone regeneration in a critical size defect model in the rat femur [8]. Interestingly, this study showed that the smallest analyzed granules led to a significantly improved bone healing compared to bigger granules accompanied with an increased number of M1 macrophages and an increased release of proangiogenic factors. Altogether, these observations underline the theory that suggested the involvement of proinflammation in both processes the biodegradation of bioceramics and the implant bed vascularization.

However, it is still unclear which influence the granule size has on the induction of multinucleated giant cells (MNGCs) that have been manifoldly found within the implantation bed of BSM [8,9]. This cell type has been identified to be a key regulator of the inflammatory tissue response, especially to a BSM, as it is involved in the degradation of biomaterials and also an influencing factor of their tissue integration [10,11]. Thus, it has been described that MNGCs can guide either the complete biodegradation of a biomaterial or its fibrotic encapsulation.

Driven by this question, the present study was conducted focusing on the influence of the granule size of synthetic BSM onto the inflammatory tissue response with special focus on the induction of pro- and anti-inflammatory macrophages and their ratio, as well as the induction of their fusion into MNGCs. Finally, the focus was on the analysis of the tissue integration behavior of the differently sized BSM granules. Therefore, three BSM with different granule sizes were implanted subcutaneously for 10, 30, and 60 days into the subcutaneous connective tissue of BALB/c mice. After explantation, previously published histological, histopathological and histomorphometrical procedures were applied [12,13].

## 2. Materials and Methods

### 2.1. Bone Substitute Materials

The bone substitute granules analyzed in the present study were synthetized based on the technology of hydroxyapatite (HA)-forming calcium phosphate cements (CPCs) that have already been described in different publications (InnoTERE GmbH, Radebeul, Germany) [14,15]. In brief, round granules with three different diameters (Table 1) were produced based on a cement powder consisting of 60% alpha-tricalcium phosphate (prepared by solid-state sintering of calcium carbonate and calcium hydrogen phosphate at a calcium:phosphate ratio of 1.45 at 1300 °C in air), 6% dicalcium phosphate anhydride, 10% calcium carbonate, and 4% precipitated HA as already described [14,15]. Characterization of the granules was also performed by the same group [15,16]. The spherical surface area to volume ratio was calculated as followed (*r* = radius):

(1) $$surface\;area\;to\;volume\;ratio=\frac{4\pi r^{2}}{\frac{4}{3}\pi r^{3}}=\frac{3}{r}$$

### 2.2. In Vivo Study

The in vivo study was initially approved by the local Ethical Committee (Faculty of Medicine, University of Niš, Niš, Serbia) based on the approval of the Veterinary Directorate of the Ministry of Agriculture, Forestry and Water Management of the Republic of Serbia (number of approval: 323-07-00278/2017-05/3 from 13 July 2017) and was carried out at the Faculty of Medicine (University of Niš, Serbia).

The study included 45 male BALB/c mice, 2-3 months old, that were randomly divided in 3 different groups, i.e., small granules (S), medium granules (M), and big granules (L) (Table 1), with *n* = 5 animals per group and time point (10, 30, and 60 days). The animals were kept under standard conditions, i.e., standard macrolon cages, standard diet, access to water *ad libitum*.

The implantation procedure was conducted as previously described [12,13,17,18]. In brief, a subcutaneous pocket was bluntly incised after anesthesia via an intraperitoneal injection with 10 mL ketamine (50 mg/mL) combined with 1.6 mL xylazine (2%) within the subscapular region of the animals. The bone substitute granules were implanted into the subcutaneous pocket that was sutured after the implantation.

At the end of the implantation periods the experimental animals were euthanized via an overdose of the anesthetics and the tissue including the implantation area was excised. Afterwards, the explants were fixed with a 4% formalin solution (Carl Roth, Karlsruhe, Germany) for 24 h followed by further histological preparation.

### 2.3. Histological Workup and Staining Methods

The sample preparation included an initial cutting of the explants in the middle and decalcification in 10% Tris-buffered EDTA (Carl Roth, Karlsruhe, Germany) for 10 weeks. Subsequently, the tissue was dehydrated in a series of graded ethanol solutions and xylene followed by embedding in paraffin. For the further preparation of histological and immunohistological stainings the embedded tissue was sectioned by means of a microtome (SLEE, Mainz, Germany) in 4 µm thick slices.

The further workup included the preparation of the following (immuno-) histochemical stainings: (a) hematoxylin and eosin (Morphisto, Frankfurt am Main, Germany) and (b) Masson Goldner in accordance the manufacturer information. Moreover, two sections were used to conduct the immunohistochemical detection of M1- and M2-macrophages. Briefly, the sections were deparaffinated by xylene and rehydrated in a series of alcohol solutions. The antigen retrieval was performed by an EDTA solution at 95 °C for 20 min, followed by 10 min incubation with blocking solution (Zytomed, Berlin, Germany). M1-macrophages were stained by using a monoclonal anti-CD11c antibody (abcam, Cambridge, UK) and M2-macrophages by using a monoclonal anti-CD163 antibody (abexxa, Arlington, TX, USA). The specific primary antibody incubated for 1 h at room temperature on the section. Afterwards, the polyclonal secondary antibody (Zytomed, Berlin, Germany) incubated for 15 min on the section. The visualization of the antibody detection was affected by application of the Permanent AP-Red System (Zytomed, Berlin, Germany) and counterstaining with hemalum.

### 2.4. Analysis Methods

The histopathological analysis was performed on basis of a previously described protocol via a light microscope (Axio.Scope.A1, Zeiss, Oberkochen, Germany) [19]. In brief, this examination focused on the occurrence of the following parameters: fibrosis; hemorrhage; necrosis; vascularization; and the presence of neutrophils, lymphocytes, plasma cells, macrophages, and multinucleated giant cells. Microscopic images were made by means of a connected microscope camera (Axiocam 305 color, Zeiss, Oberkochen, Germany) in combination with a computer system running the ZEN Core 3.0 (Zeiss, Oberkochen, Germany) connected to the microscope.

The histomorphometry was conducted based on a procedure described by Barbeck et al. [12]. Briefly, the immunohistochemically stained slices were digitized by a scanning microscop (PreciPoint M8, Freising, Germany) with a maximal edge of 26.000 pixels. The total scans were analyzed using the software Image J. The polygon section tool was used to mark the tissue surrounding the implanted biomaterial. The stained cells were then accentuated using a special plugin developed by Lindner et al. [13] and calculated as cells/mm^2^. Furthermore, the multinucleated giant cells that were associated with the implant were calculated manually and related to the area of the implantation bed (MNGC/mm^2^).

### 2.5. Statistics

GraphPad Prism 9 (GraphPad Software Inc., La Jolla, CA, USA) was used for the statistical analysis. The data were analyzed by an analysis of variance (ANOVA) and a following LSD post hoc test to allow for the comparison of the data from the different study groups. Thereby, data were marked as significant if *p*-values were less than 0.05 (* *p* ≤ 0.05) and highly significant if *p*-values were less than 0.01 (** *p* ≤ 0.01) or less than 0.001 (*** *p* ≤ 0.001) and graphed as mean ± standard deviation.

## 3. Results

### 3.1. Histopathological Results

**Figure 1:**
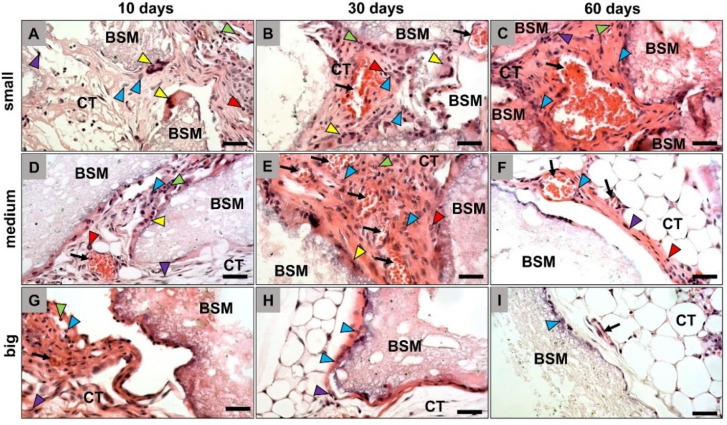
Exemplary histological images of the tissue reactions to the differently sized bone substitute materials (BSM), i.e., the small BSM granules (**A**–**C**), the medium-sized BSM granules (**D**–**F**), and the big BSM granules (**G**–**I**). CT = connective tissue, blue arrowheads = macrophages, yellow arrowheads = multinucleated giant cells, purple arrowheads = fibroblasts, red arrowheads = eosinophils, green arrowheads = neutrophils, black arrows = blood vessels (HE-staining, 400× magnifications, scalebars = 50 µm).

The histopathological analysis revealed that all granule sizes were detectable within the subcutaneous connective tissue inducing a material-related inflammatory tissue response at day 10 *post implantationem* (Figure 1). In all groups, a granulation tissue including mainly macrophages and fewer numbers of granulocytes and fibroblasts was observable, as well as single vessels were observable at this early study time point. Furthermore, mainly macrophages and single multinucleated giant cells (MNGCs) were found at the material surfaces (Figure 1A,D,G).

At day 30 *post implantationem*, a cell- and vessel-rich granulation tissue including same cell types as mentioned before was found within the intergranular interspaces in the groups of the small and medium-sized BSM granules (Figure 1B,E). Still, macrophages and MNGCs were detected at the granule surfaces. In contrast, only a thin cell layer was found at the granule surfaces of the big BSM granules that was also mainly consisting of macrophages and MNGCs, while the intergranular spaces were mainly filled out by fatty tissue (Figure 1H).

At day 60 *post implantationem*, the same granulation tissue with the aforementioned composition was only found within the implantation beds of the small BSM granules including also mainly macrophages and MNGCs at the granule surfaces (Figure 1C). In the group of the medium-sized granules only a very thin tissue layer was found at the granule surfaces mainly composed of macrophages, fibroblasts, and single MNGCs (Figure 1F). Thereby, the intergranular spaces were most often filled up with fatty tissue at this study time point. In the group of the big BSM granules only single cells that were mainly macrophages were observable at the material surfaces, while the implantation beds were also mainly including fatty tissue at this study time point (Figure 1I).

**Figure 2:**
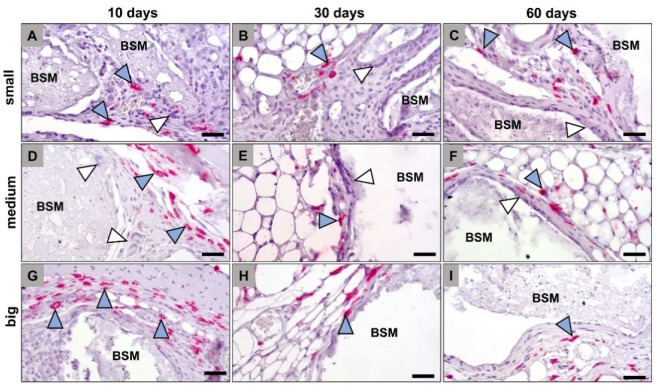
Exemplary histological images of anti-inflammatory (CD163-positive) macrophages (blue arrowheads) within the implant beds of the differently sized bone substitute materials (BSM), i.e., the small BSM granules (**A**–**C**), the medium-sized BSM granules (**D**–**F**), and the big BSM granules (**G**–**I**). CT = connective tissue, white arrowheads = CD163-negative multinucleated giant cells (CD163-immunostainings, 400× magnifications, scalebars = 50 µm).

**Figure 3:**
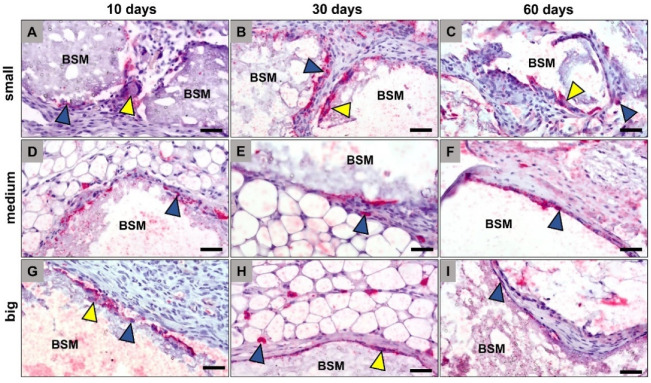
Exemplary histological images of pro-inflammatory (CD11c-positive) macrophages (blue arrowheads) and multinucleated giant cells (yellow arrowheads) within the implant beds of the differently sized bone substitute materials (BSM), i.e., the small BSM granules (**A**–**C**), the medium-sized BSM granules (**D**–**F**), and the big BSM granules (**G**–**I**). CT = connective tissue (CD11c-immunostainings, 400× magnifications, scalebars = 50 µm).

The histological analysis of the occurrence of anti- and proinflammatory macrophage and MNGC subtypes within the implantation beds of the three BSM types revealed that comparable numbers of both CD163- and CD11c-positive cells were found in all groups at any time point (Figure 2 and Figure 3). Thereby, it was observable that the CD163-positive cells that were solely mononuclear have been found only within peripheral regions of the granules’ implantation beds (Figure 2). Thus, both the macrophages and the MNGCs at the granule surfaces were CD163-negative (Figure 2). In contrast, the CD11c detection showed that mainly the material-adherent cells, i.e., both the mononuclear and the multinucleated cells, were expressing this proinflammatory molecule (Figure 3).

### 3.2. Histomorphometrical Results

**Figure 4:**
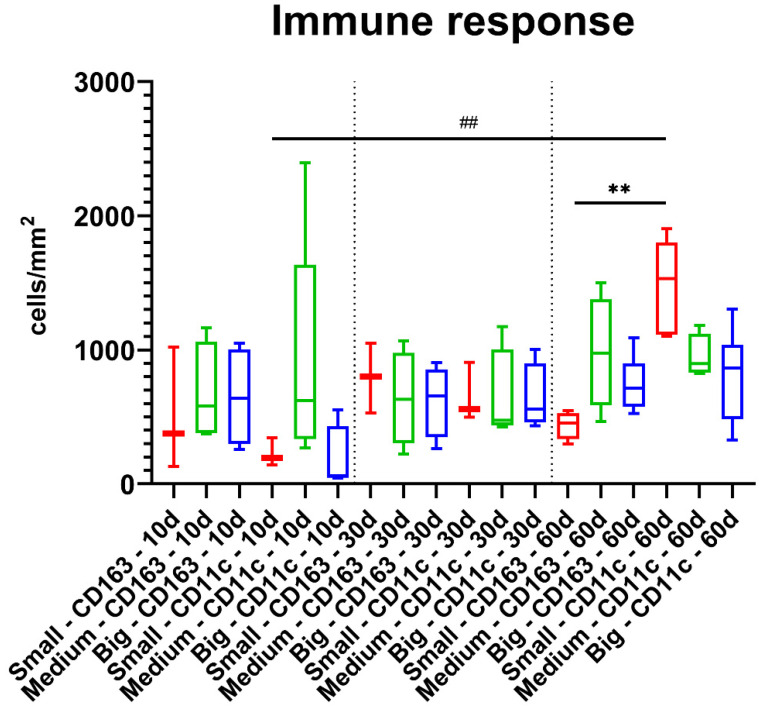
Histomorphometrical results of the measurements of macrophages subtypes (intraindividual: ** *p* < 0.01, interindividual: ^##^ *p* < 0.01). Red: small granules, green: medium-sized granules, and blue: big granules.

The results of the histomorphometrical measurements showed that no significant differences were found between the values of the M1- and M2-macrophages between the three different study groups at day 10 *post implantationem* (Figure 4 and Table 2). Thereby, very comparable numbers of CD163-postivie macrophages were found, while in case of the CD11c-positive cells a trend towards a higher occurrence was found in the group of the medium-sized bone substitute granules (Figure 4 and Table 2). The lowest numbers of CD11c-positive macrophages were found in the group of the big granules, while comparably low values were detectable in the group of the small bone substitute granules at this early study time point (Figure 4 and Table 2).

At day 30 *post implantationem*, still comparable numbers of both CD163- and CD11c-positive macrophages were found in all study groups without any trends towards a pro- or anti-inflammatory tissue reaction (Figure 4 and Table 2).

At day 60 *post implantationem*, still comparable numbers of both macrophage subtypes were found within the implant beds of all study groups (Figure 4 and Table 2). However, higher numbers of CD11c-positive macrophages were detected in the group of the small bone substitute granules that were significantly higher (** *p* < 0.01) compared to the numbers of CD163-positive cells in this group (Figure 4 and Table 2). Furthermore, the numbers of CD11c-positive macrophages at day 60 were significantly higher (** *p* < 0.01) compared to the numbers of this macrophage sub-form at day 10 *post implantationem* (Figure 4 and Table 2).

**Figure 5:**
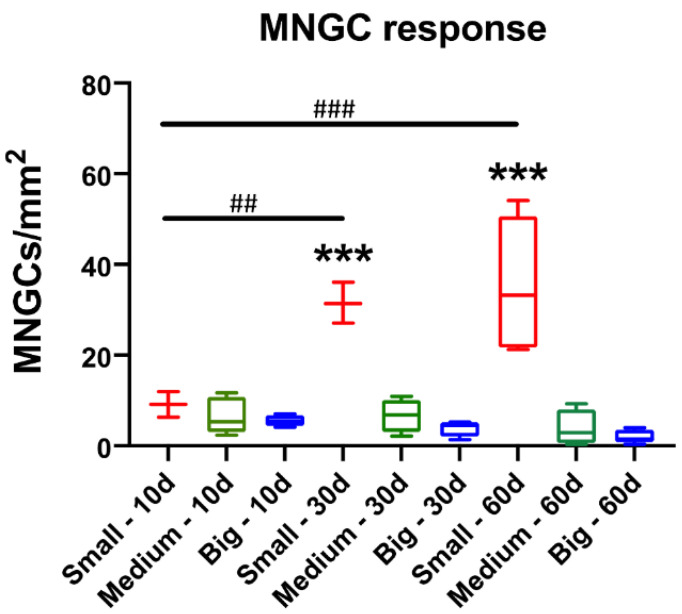
Histomorphometrical results of the occurrence of multinucleated giant cells (MNGCs) (intraindividual: *** *p* < 0.001, interindividual: ^##^ *p* < 0.01 and ^###^ *p* < 0.001). Red: small granules, green: medium-sized granules, and blue: big granules.

Additionally, the histomorphometrical analysis of the MNGC induction showed that no significant differences between the numbers of MNGCs in the three study groups were measured at day 10 *post implantationem* (Figure 5 and Table 3). However, the trend showed that the MNGC numbers decreased with the increase of the granule size at this early time point. At day 30 and day 60 *post implantationem*, the measurements showed that significantly higher MNGC numbers (*** *p* < 0.001) were detected in the group of the small BSM granules compared to the values in both other study groups that did not significantly differ (Figure 5 and Table 3). However, it was still observable that the MNGC numbers were decreasing with the granule diameter rise.

Moreover, the analysis revealed that the MNGC numbers in the group of the small BSM granules significantly increased over time (** *p* < 0.01 and *** *p* < 0.001) (Figure 5 and Table 3). In contrast, the MNGC numbers in the groups of the medium-sized and big BSM granules remained at the same level over the complete stud period, while in both groups a trend towards an MNGC decrease from day 10 to day 60 *post implantationem* was observable. Thereby, the trend seems to be more pronounced in the group of the big BSM granules (Figure 5 and Table 3).

## 4. Discussion

In the present study, the effect of granule size on the biocompatibility of synthetic BSM was investigated. Particular attention was paid to the quantification of M1 and M2 macrophages as well as multinucleated giant cells (MNGCs). This study was conducted to clarify the question whether granule size has an influence onto the induction of macrophage subtypes and MNGC formation. Both cell types have been identified to be a key regulator of the inflammatory tissue responses to biomaterials and especially to BSM [10,11].

Initially, the histopathological results showed that the granule size has a major influence on the host tissue reaction, especially on the integration pattern and also on the long-term cell-material interactions. It was observed that a layer of fibrous tissue surrounded the big and medium-sized BSM at 30 and 60 days *post implantationem*. In contrast, functional granulation tissue was detected at the material surfaces in the group of the small granules even at 60 days *post implantationem*. In addition, compared to the medium and big granules, an increased occurrence of macrophages and MNGCs was observed which have already shown to be mainly responsible for the intra- and extracellular degradation and phagocytosis of biomaterials [20]. Thus, it is assumable that the observed functional granulation tissue seems to allow for the ongoing degradation of the small BSM granules as newly recruited phagocytes can still be observed in the implantation beds of the small granules at this late study time point, which are known to express lytic enzymes that degrade the granules [21]. In contrast, the results of the histopathological analysis revealed that the medium-sized and big BSM seemed to be completely passivated at 60 days *post implantationem*. This was concluded based on the lower presence of the cell types involved in the biodegradation.

Thereby, these observations support the work of Barbeck et al. that stated three different material-mediated pathways of the involvement of MNGCs after BSM application [22]:

(i) scenario 1: complete resorbability of a BSM mediated by mononuclear and multinucleated phagocytes,
(ii) scenario 2: MNGC-mediated fibrous encapsulation of a biomaterial without mediation of a material-associated further healing process, and
(iii) scenario 3: persistence of phagocytes at the surfaces of biomaterials that may support preservation of bone tissue within the implantation bed of a BSM by continuous expression and secretion of molecules involved in the bone healing process such that no resorption is processed by these cells.

Based on the observations of the present study, it can be concluded that the small granules seem to be completely degradable (in accordance with scenario 1), while both the medium-sized and the big granules lead to a fibrous encapsulation without an ongoing cellular degradation (in accordance to scenario 2). Thus, only the tissue reactions to the small granules are in line with the concept of “creeping substitution” [23,24]. This term was introduced by Phemister stating that transplanted bone is initially invaded by vascular granulation tissue, causing the old bone to be resorbed and subsequently replaced by the host with newly formed bone [24]. This concept has been transferred also to synthetic bone substitute materials that have shown to be resorbed mainly by phagocytes, i.e., macrophages and MNGCs [25]. Thus, the “fate” of the small granules seems to be their full bio-resorption while serving as osteoconductive scaffolds, while both the medium- and large-sized BSMs seem to be delineated and may not be fully functional in the course of material-mediated bone regeneration.

The question of the underlying molecular or cellular reasons for the encapsulation of the granules in these two groups still arises as only poor knowledge about these mechanisms exists until now. It must again be mentioned that the analyzed BSM granules only differed in the granule diameters, while all other material factors such as chemical composition are exactly the same. Moreover, the BSM granules examined in the present study are hydroxyapatite-forming, which was reported by Heinemann et al. via X-ray diffraction analysis and scanning electron microscopic imaging [16]. In this context, it has to additionally be mentioned that HA-based materials are resorbed (very) slowly due to both their low dissolution behavior but also based on the low cellular degradability [26]. Interestingly, Okuda and colleagues that analyzed hydrothermally synthesized pure calcium-deficient HA-based BSM hypothesized that this material was not continuously recognized and resorbed by osteoclasts, which is in line with the actual results—especially with the histopathological results and the histomorphometrical measurement values [27].

One explanation of this phenomenon is that even the phagocytosis capacity of the MNGCs is restricted in case of the granules with a higher diameter. An equivalent process has been shown in case of mononuclear phagocytes or macrophage, which phagocytized granule fragments of a biphasic BSM with a mean diameter of 0.6 µm due to their restricted membrane capacity [6]. However, in this previous in vivo study no measurement of the phagocytosis of MNGCs was possible due to technical reasons. It is conceivable that the granules in these two groups are encapsulated because the phagocyte system or its cellular elements are overwhelmed with the cell-based degradation. Interestingly, the degradation of bigger granules such as the analyzed materials of the present study are primarily degraded via “extracellular” processes within a subcellular compartment built between the cell body of a MNGC and the biomaterial, while the phagocytosis by MNGC takes place after detaching of small(er) material subunits [6]. Thereby, the phagocytosis capacity of MNGCs is still unknown and also the present study could not elucidate this important material factor, so that more studies involving smaller biomaterials are necessary. However, the data lead to the conclusion that both bigger granule types lead to a succumbing of the degradation due to exceeding the capacity of MNGCs. In this context, it is also thinkable that both the chemical basis and the size of the materials may combinatorically have contributed to this result even due to the aforementioned well-known resorbability of HA-based BSM.

However, this result does not mean that the biomaterials are excluded for all applications or indications. On the one side, a comparable phenomenon has been observed in case of xenogeneic BSM whose osteoconductive potential has already manifoldly been proven in preclinical and clinical studies [28]. On the other side, the study period of the present study as well as the implantation side may lead to a restricted meaningfulness. Thus, the subcutaneous implantation model allows only for statements about the basic tissue compatibility of medical devices such as the analyzed BSM and may not allow for a “transfer” of the data into the real clinical situation or the bony microenvironment. However, it has also shown that the results of different preclinical studies using this implantation model could be transferred to the clinical situation, whose investigability is often restricted due to the harvest of related biopsies [28].

In the context of foreign body reaction (FBR), the macrophage-induced immune response has a major impact on the integration of biomaterials within a defect side [20,29]. In the present study, the positive area of M1 and M2 macrophages induced by the three BSM were quantified to compare the overall inflammatory “direction” of the tissue reaction. The two macrophage subtypes originate from monocytes that migrate into the material-induced inflammatory tissue, where they differentiate into both inflammatory subtypes depending on the physicochemical material properties [30]. The proinflammatory M1 macrophages possess, among other things, the ability to phagocytose foreign material in the body [30]. The anti-inflammatory M2 macrophages possess, among other properties, the ability to trigger processes like cell proliferation and are thereby known to support regeneration of the damaged tissue [31,32,33]. In case of the analyzed BSM granules, it can be seen that the anti-inflammatory M2 response remains constant over the study periods regardless of the granule size. Similarly, the M1 activity was also constant for the medium and big granules. However, for the small granules, the M1 activity increases significantly at day 60 *post implantationem* compared with both M1 activity after 10 days and M2 activity after 60 days. Furthermore, CD11c-positive M1 macrophages were mainly observed at the material surfaces of the analyzed BSM. In contrast, the CD163-positive M2 macrophages were predominantly found within the surrounding granulation tissue and not on the surface of the implant.

The fact that M1 macrophage activity increased in the group of the small granules after 60 days does not equate to deteriorated biocompatibility of the granules but underlines the results obtained via histopathology. As mentioned above, the key role of the proinflammatory M1 macrophages is the phagocytic activity towards the BSM. Thus, the histomorphometrical data also indicate that the small BSM granules underwent a higher biodegradation—although no differences of the M1 or M2 macrophage numbers compared to the other groups were measured. These conclusions are also supported by the results of quantification of MNGCs as their number was significantly higher in the group of the small BSM granules compared to the other granule sizes at 30 and 60 days *post implantationem*. This result additionally suggests that the phagocytosis activity in the group of the small BSM granules is still continuing.

In this context, a previous in vitro study by our group had already shown that the granule size has an influence on cytokine expression, when they are co-cultured with primary monocytes [34]. It was found that BSM granules based on beta-tricalcium phosphate (ß-TCP) with a diameter > 500 µm induced a significantly higher cytokine expression of interleukin 10 (IL-10) and IL-12 leading to the assumption of a reduced MNGC formation. However, the results of the present study showed the small granules induced a higher MNGC number. These results are thus not consistent with the previous in vitro results but are comparable with that shown by different preclinical in vivo studies that showed similar MNGC numbers induced by small BSM granules [6,35,36]. Thus, a discrepancy between the in vitro and the in vivo results can be noticed. In this context, it was shown in an in vitro study conducted by Shrivastava and colleagues that the addition of IL-10 resulted in reduced MNGC formation [37]. Contrastingly, the addition of IL-4 led to an increased MNGC formation. Thus, it can be concluded that some cytokines have an overriding role in MNGC formation such as IL-4 and IL-10 [34,37]. Altogether, these data show that those in vitro results still cannot replace in vivo studies due to their limitations. Other in vitro investigation of the influence of granule size of BSMs focuses mostly on osteogenesis and behavior of seeded osteoblasts, not on the induction of macrophage fusion or degradation [38,39]. However, many groups have investigated granule size in vivo with different implantation models. Barbeck et al. and Ghannati et al. reported that smaller particle size of BSMs induced higher vascularization, macrophage fusion, and TRAP activity. All observations point to faster degradation behavior [6,35]. Interestingly, in induced bone defects in vivo, Jung et al. did not report any significant difference in bone regeneration between the variable particle sizes [36]. However, this can be due to the short time period of the study. In contrast, other groups reported increased bone regrowth with the smaller BSM particles in different animal models, and with extended implantation time [40,41]. This could be explained by the increased activity of MNGCs, as they can increase vascularization and enhance new bone growth via the concept of creeping substitution [23,24]. Evidently, Malard et al. reported an increased occurrence of MNGCs surrounding the smaller particles and in the same study, the smaller size particle exhibited a higher degradation rate [9].

As the BSM granules did not differ in any properties except for size, it can be concluded that a crucial factor for the induction of MNGC is the increased surface area-to-volume of the granules. Based on this, two possible modes of action are conceivable:

(1) The surface-to-volume ratio increases with decreasing particle size, which means that a much larger surface area is “available” in case of small granules (Table 1). Thus, the increased surface area may lead to increased “binding sites” for phagocytes. To further elaborate on this, the surface of the biomaterial (in our case the BSM) is initially adsorbed with small molecules (e.g., water molecules, ions, etc.) [29,42]. Which in turn increases forces like Van der Waal, hydrophobic/hydrophilic forces and so on. This dynamic increase of forces would attract the different types of blood proteins as they are complex molecules with different forces and active sites. By then ‘competitive’ adsorption occurs and the most fitting protein will adhere to the surface [42]. Macrophages are adhering-dependent cells, which means that this cell type even in the context of the foreign body response to biomaterials survive only if they can attach to a material. If prompted, the macrophages will fuse to form MNGCs [29,43]. The fusion always requires certain molecular mediators (cytokines), as well as proper surface adhesion [44]. The more surface area a biomaterial provides, the more macrophages and also MNGCs can attach.
(2) Ion-induced MNGC formation is present in case of the small BSM granules. It is well known that Calcium phosphate-based biomaterials release Ca^2+^- and PO_4_^3−^-ions over time [45,46]. Calcium ions bind to membrane associated soluble N-ethylmaleimide-sensitive-factor attachment receptor (SNARE) complexes found on secreting cells [44]. The binding results in the formation of channels that release water, which leads to a destabilization of the cell membrane. Finally, destabilization leads to membrane fusion [44,47]. Thus, it is conceivable that the increased surface area-to-volume leads to increased release of calcium ions, encouraging the fusion of the adherent macrophages.

The clinical application of BSM always depends on the type of application and the patient collective. Elderly patients have poorer bone quality and a limited healing rate [48]. This is often caused by osteoporosis, which can be triggered by diabetes, or osteopenic syndrome, which is more common in postmenopausal women. These and other factors promote a higher rate of bone fractures in the elderly [49]. Here, the resorption rate can have a major impact on the healing process, for example, to support bone regeneration through faster resorption. The resorption of BSM plays a crucial role in the type of application. In many applications, such as alveolar bone augmentation prior to prosthetic implantation, BSMs are used to fill the space that is gradually filled with new bone [50,51]. In other applications, BSMs are again used to replace bone that is not present, such as in bone filling in the sinus [52]. In the first case, for example, smaller granules can be chosen to degrade over time to be filled in turn with new bone. In the case of bone augmentation, where BSMs are implanted in sites where no bone is naturally present, bigger granules could be used with the aim of passivation. Further studies are needed to confirm the accuracy of both theories.

Based on the present data, it can be concluded that the integration as well as the degradation behavior of synthetic bone substitutes can be influenced by granule size. These findings can moreover substantiate different previous observations made in preclinical and clinical studies via molecular data. Finally, they show that this material factor allows clinicians such as dentists more insights into the biological consequences when faced with the choice of which particle size of a bone substitute to select.

## Figures and Tables

**Table 1:** Overview of the granulate diameters.

Granulate	Granulate Diameter [mm]	Surface Area to Volume Ratio [mm^−1^]
Small	0.355–0.500	12–16.9
Medium	0.500–1.000	6–12
Big	1.000–2.000	3–6

**Table 2:** Results of the histomorphometrical measurements of macrophages subtypes.

Timepoint/Size	Small		Medium		Big	
	CD163+	CD11c+	CD163+	CD11c+	CD163+	CD11c+
Day 10	508.6 ± 459.2	226.8 ± 104.7	793.6 ± 259.8	654.9 ± 221.1	435.9 ± 101.6	1473.0 ± 353.3
Day 30	692.6 ± 354.1	912.7 ± 860.3	638.9 ± 350.9	637.9 ± 357.6	981.7 ± 422.0	949.3 ± 161.9
Day 60	646.7 ± 372.7	180.6 ± 250.4	619.8 ± 268.7	638.2 ± 251.4	744.5 ± 209.4	807.5 ± 343.1

**Table 3:** Results of the histomorphometrical measurements of MNGCs.

	Small (MNGCs/mm^2^)	Medium (MNGCs/mm^2^)	Big (MNGCs/mm^2^)
Day 10	9.15 ± 2.82	6.59 ± 4.01	5.54 ± 1.25
Day 30	31.53 ± 4.49	6.67 ± 3.64	3.94 ± 1.77
Day 60	35.60 ± 14.73	3.87 ± 3.98	1.96 ± 1.42

## Data Availability

All data are included in the manuscript.
